# Supplementary material for: Effects of Message Framing and Information Source on Consumers’ Attitudes toward an Amino Acid-Based Alternative Meat Curing System
Source: Foods. 2023 Apr 5;12(7):1535. doi: 10.3390/foods12071535 (PMC10094039; doi:10.3390/foods12071535)
Supplement: Supplementary file 1 [file foods-12-01535-s001.zip › foods-2244574-supplementary narrative video script; analytical video script.pdf]

Narrative-script

Sodium nitrite keeps the bacon that compliments our scrambled eggs sizzling, our holiday hams pink and juicy, and our grilled hotdogs just right at the Texas A&M tailgates. Most meat products are cured by the direct addition of sodium nitrite, which produces the distinctive cured color, satisfying flavor, and long shelf-life of our favorite cured meat products.

However, people are worried about their health when consuming conventionally cured meat. Concerns around synthetic nitrite, paired with worry about carcinogenic compounds, contribute to demand for alternative meat curing systems. Although scientists have explored alternatives like the indirect addition of sodium nitrite, through high nitrite sources like vegetable powder, no single ingredient exists to replace the color, flavor, shelf life, or safety of conventional curing

But, thanks to the hard work and dedication of meat scientists at Texas A&M, cured products like the pepperoni on your pizza or bologna in your sandwich, could potentially be cured without the direct, or indirect, addition of sodium nitrite. The possibility exists to add L-arginine to meat products, activating the endothelial nitric oxide synthase system that is naturally found in meat to produce nitric oxide and residual nitrite. These are the same compounds our body forms when we eat our vegetables, like our parents always told us to.

This amino acid-based alternative meat curing system could provide us with a more natural alternatively cured meat product. There's potential to capitalize on an enzyme already found in meat, using an amino acid we need, to produce the same shelf-life that keeps our summer sausage good all summer long, the familiar array of colors on a charcuterie board at an elegant wine night, and tasty flavors and unique textures we appreciate about cured meat classics eaten at sporting events, holiday dinners, and backyard barbecues.

Analytical Script

Cured meat products include, but are not limited to, sausages, bacon, hams, jerky, dried meat products, and fermented products. Currently, the primary method for curing meat is by the direct addition of sodium nitrite. Sodium nitrite is a highly reactive crystalline salt functioning as an oxidizing, reducing, or nitrosylating agent. When added to meat, it serves as an antimicrobial and antioxidant, contributing to a longer shelf life. Sodium nitrite generates nitric oxide which, when bound to muscle myoglobin, produces the cured pink color. The indirect addition of sodium nitrite, through high nitrite sources like vegetable powder, have been explored as alternatives to conventional meat curing. However, no single ingredient exists that replaces the color, flavor, shelf life, and safety of curing meat with sodium nitrite.

Previous research trials indicate that an amino acid-based alternative meat curing system can produce cured meat color and provides an antimicrobial and antioxidant effect similar to conventional curing. The addition of L-arginine, an amino acid found in muscle cells, can activate the endothelial nitric oxide synthase system, also called eNOS, which generates nitric oxide and residual nitrite. Meat scientists investigated the feasibility of adding L-arginine to post rigor skeletal muscle, or meat, and evaluated its impact on the eNOS system's ability to generate nitric oxide and residual nitrite.

Results indicated that L-arginine concentrations of 1000 to 4000 parts per million for beef, 1000 to 3000 parts for million for pork, and 1000 to 2000 parts per million for poultry generated residual nitrite values comparable to sodium nitrite treated beef, pork, and poultry samples. This indicates that an amino acid-based alternative curing system has the potential to replace synthetic sodium nitrite used in conventional curing and the indirect addition of sodium nitrite in alternative meat curing systems.
