# Supplementary material for: Effects of Message Framing and Information Source on Consumers’ Attitudes toward an Amino Acid-Based Alternative Meat Curing System
Source: Foods. 2023 Apr 5;12(7):1535. doi: 10.3390/foods12071535 (PMC10094039; doi:10.3390/foods12071535)
Supplement: Supplementary file 1 [file foods-12-01535-s001.zip › foods-2244574-supplementary Texas A&M curing meat without added nitrites - AgriLife Today.pdf]

(https://agrilifetoday.tamu.edu/)

Texas A&M meat scientist developing 'no nitrite-added' cured meats

New technique would use an amino acid instead of sodium nitrite

February 20, 2023

Imagine your favorite cured meat like beef jerky, pepperoni or bacon without any added sodium nitrite from any source currently necessary for color and shelf life. Wes Osburn, Ph.D., is doing exactly that.

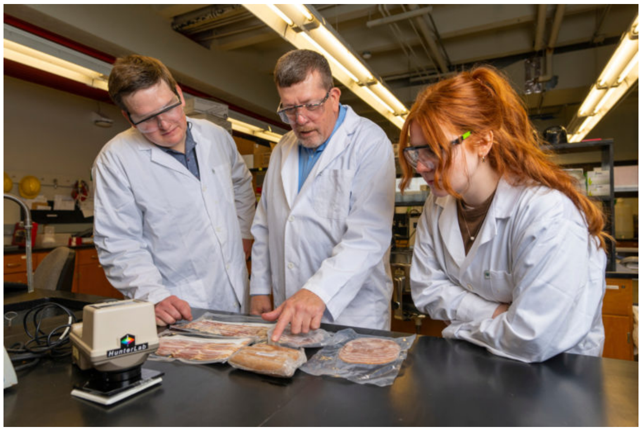

At center, Wes Osburn, Ph.D., Texas A&M University meat scientist, is working in his lab with students Tanner Wright and Arlie Reeves on a new no nitrite-added cured meat system. (Texas A&M AgriLife photo by Michael Miller)

Osburn, associate professor in meat science in the Texas A&M College of Agriculture and Life Sciences (https://aglifesciences.tamu.edu/) Department of Animal Science (https://animalscience.tamu.edu/), has set out to find an innovative method to generate the nitric oxide and residual nitrite needed to cure meat and poultry products, but without the addition of natural or synthetic nitrite sources.

Conventional curing of most processed meat products involves adding sodium nitrite to meat to preserve it and reduce the potential growth of bacteria like Clostridium botulinum or perfringens during cooking and chilling.

Curing meat

Osburn has been researching the idea of a novel amino acid alternative curing system for meat for many years. In 2022, he received a $500,000 U.S. Department of Agriculture (https://www.usda.gov/)–Agriculture and Food Research Initiative (https://www.nifa.usda.gov/grants/programs/agriculture-food-research-initiative-afri) grant to determine the feasibility of adding amino acids (https://cris.nifa.usda.gov/cgi-

Website Feedback

bin/starfinder/0?
path=fastlink1.txt&id=anon&pass=&search=R=94915&format=WEBLINK) to activate the nitric oxide synthase system.

Joining him in the research are Department of Animal Science faculty members Rhonda Miller, Ph.D., professor in meat science; Guoyao Wu, Ph.D., Distinguished Professor, University Faculty Fellow and Texas A&M AgriLife Research Faculty Fellow in animal nutrition; Sapna Chitlapilly Dass, Ph.D., assistant professor in microbial ecology and microbiome interactions; and Chris Kerth, Ph.D., associate professor in meat science; along with Ranjith Ramanathan, Ph.D., professor in meat science, Oklahoma State University.

While curing meat with synthetic sodium nitrite is safe and efficient, the process has long been associated with cancer concerns. In response to these concerns, meat processors have developed "uncured" meat products that contain "no added nitrates or nitrites except those naturally occurring."

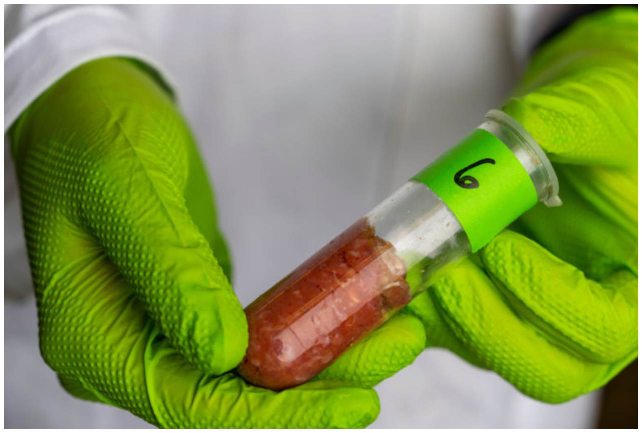

Using naturally occurring nitrites and nitrates, a team led by Wes Osburn, Ph.D., Texas A&M University meat scientist, is working to make sure they can obtain the pink color of cured meats. (Texas A&M AgriLife photo by Michael Miller)

This alternative curing system uses vegetable powder derived from celery as a natural source of nitrite to cure meat products. But these products may result in a detectable vegetable taste to the meat and a less favorable cured meat, or pink color, Osburn said.

Knowing that nitrites and nitrates occur naturally in humans and in some foods, Osburn believes it is possible to add an amino acid, L-arginine, to the meat that activates the nitric oxide synthase, NOS, enzyme contained in it. The NOS enzyme converts L-arginine to nitric oxide, NO, and another amino acid, L-citrulline.

The nitric oxide molecule creates the characteristic cured pink color associated with cured meats. Also, two nitric oxide molecules can combine to form nitrite, which serves as an antioxidant and antimicrobial to enhance product shelf life and safety.

Website Feedback

Osburn said he was listening to a medical doctor friend, Dr. Nathan Bryan, speak about the endothelial nitric oxide synthase, eNOS, system in the human body and how it uses L-arginine to generate nitric oxide, which enhances circulation and blood flow. That is when the connection clicked, and he wondered if the eNOS enzyme could be activated by adding L-arginine to generate nitric oxide and cure meat.

"It could be that this approach is actually a 'natural curing process' by adding an amino acid to an enzyme whose primary function is to generate nitric oxide," Osburn said. "I'm not adding any nitrates or nitrites."

Utilizing natural amino acids

Osburn said adding the amino acid L-arginine will activate the nitric oxide synthase system to naturally generate nitric oxide and nitrite to cure meat and poultry products.

This novel amino acid-based alternative curing method is expected to eliminate the need for direct or indirect addition of sodium nitrite in cured meat products. Their research will allow for a better understanding of the interconnected biochemical mechanisms contributing to the functionality of the NOS system.

The research team plans to develop processing and operating procedures for meat processors to effectively use the NOS system to cure meat and poultry products consistently and predictably.

Osburn said they must validate the process's feasibility and ensure it works, as well as make sure it works across species and products within species under all kinds of conditions. He said it will take a while to run the research on the different types of cured beef, pork and poultry products.

"There are differences in the amount of the NOS enzymes within different muscle groups across various meat species," he said. "This enzyme is closely associated with the mitochondria, so there tends to be more NOS enzyme in muscles used for locomotion. There are also differences in myoglobin content (meat color pigment), so if we can generate nitric oxide via the NOS enzyme, the cured meat color may vary.

"That's what we are trying to deal with, developing a uniform cured pink color via our amino acid-based curing system so that it compares favorably with conventionally cured meats."

Website Feedback

Where's the meat?

"The question we are still trying to answer is will the NOS enzyme generate sufficient amounts of nitric oxide to develop acceptable cured meat color and enough residual nitrite to ensure that the product is safe, regardless of whether it is summer sausage or pepperoni or some other product?" Osburn said. "Think about it, if we can make pepperoni through this process, there could be a huge economic impact since we consume a lot of pepperoni here in the U.S."

He said he is developing a prototype amino acid-cured ham product that will be taken through a manufacturing sensory analysis for cured color pigment, volatile compounds, sensory and textural analysis and shelf life.

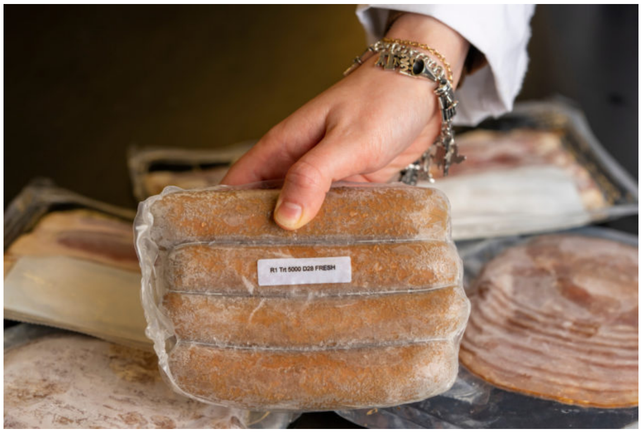

Cured meat products made without the addition of sodium nitrite are pictured at a lab of Wes Osburn, Ph.D., Texas A&M University meat scientist. (Texas A&M AgriLife photo by Michael Miller)

His research team is manipulating several factors, such as the arginine concentration, meat pH, temperature and time to determine optimal conditions for nitric oxide generation by the eNOS enzyme.

"We are in the second phase of our USDA-NIFA-funded grant," Osburn said. "We are shifting from benchtop to pilot plant production, so it is the first time we are making a commercial cured meat product and evaluating all aspects of the product from color, shelf life, aroma and sensory properties. This information will provide more evidence to show interested companies that this system works."

His research team plans to apply this new curing system to five cured products – restructured ham, beef jerky, bacon, fermented summer sausage or salami and poultry frankfurters – all products requiring different processing steps to validate that the amino acid curing system effectively cures different meat products.

Moving to the cured meat case

"If, based on the results of our research, the data indicates that our new curing system is comparable to conventionally cured products with respect to safety, shelf life and sensory attributes, then there is a great chance for industry adoption of this

Website Feedback

process," Osburn said. "This new curing system must compete favorably with the current curing system. If we can't get close to it, it'll always be a novel thing. Companies may or may not want to get on board."

Osburn said while he is getting a lot of interest from meat companies in the process, there is still a lot of research to be done, as well as some upcoming rulings by the U.S. Department of Agriculture that will determine future labeling of current alternative or "uncured" meat products.

Robert Brummett, senior licensing manager at Texas A&M AgriLife Research's (https://agriliferesearch.tamu.edu/) Intellectual Property and Commercialization (https://ipc.agrilife.org/) office, has worked with Osburn to seek intellectual property protection for the alternative meat curing process. A patent application was filed with the U.S. Patent and Trademark Office, USPTO, in December 2020.

According to Brummett, the patent application is currently being examined by the USPTO, and that process may take an additional 18 months or longer to complete. In the meantime, Osburn and Brummett are actively seeking interest from companies to license rights in the technology and/or working with Osburn in furthering the research for use of the technology by industry.

Website Feedback

-30-

Media Inquiries

Laura Muntean
laura.muntean@ag.tamu.edu
6012481891

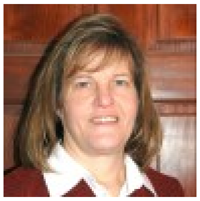
Kay Ledbetter
806-677-5608
skledbetter@ag.tamu.edu

Kay Ledbetter is communications coordinator for Texas A&M AgriLife. Additionally, she is responsible for writing news releases and feature articles from science-based information generated by the agency across the state, as well as the associated media relations.

Phone: 979.803.1287 | news@ag.tamu.edu
